# The aryl hydrocarbon receptor and the gut–brain axis

**DOI:** 10.1038/s41423-020-00585-5

**Published:** 2021-01-06

**Authors:** Andreia Barroso, João Vitor Mahler, Pedro Henrique Fonseca-Castro, Francisco J. Quintana

**Affiliations:** 1 Ann Romney Center for Neurologic Diseases, Brigham and Women’s Hospital, Harvard Medical School, Boston, MA USA; 2 Faculdade de Medicina FMUSP, Universidade de São Paulo, São Paulo, SP Brazil; 3 Broad Institute of MIT and Harvard, Cambridge, MA USA

**Keywords:** Aryl hydrocarbon receptor, Autoimmune diseases, Infectious diseases, cancer, Immunology, Inflammation

## Abstract

The aryl hydrocarbon receptor (AHR) is a ligand-activated transcription factor initially identified as the receptor for dioxin. Almost half a century after its discovery, AHR is now recognized as a receptor for multiple physiological ligands, with important roles in health and disease. In this review, we discuss the role of AHR in the gut–brain axis and its potential value as a therapeutic target for immune-mediated diseases.

## Introduction

In the 1970s, Poland et al.^1,2^ identified a potential receptor for the anthropogenic compound 2,3,7,8-tetraclorodibenzo-p-dioxin (TCDD).^3^ Two years later, the same group demonstrated that TCDD binds to this unknown receptor in hepatic cells, inducing expression of the aryl hydrocarbon hydroxylase enzyme encoded by *CYP1A1*.^4^ Those and other seminal studies by Nebert and Poland led to the identification and characterization of the aryl hydrocarbon receptor (AHR),^3–6^ a ligand-activated transcription factor with important physiological roles in health and disease.^7^

Indeed, although initial studies focused on ligands such as polycyclic aromatic hydrocarbons (PAHs), polychlorinated biphenyls (PCBs) and halogenated aromatic hydrocarbons (HAHs), it is now clear that a broad range of dietary, commensal and endogenous, ligands activate AHR.^8–11^ To date, multiple physiological and dietary AHR ligands (Table 1) have been identified, including tryptophan metabolites such as 6-formylindolo[3,2-b]carbazole (FICZ), kynurenine, indigo, indirubin, the pigment curcumin,^12^ carotenoids,^13^ flavonoids, bilirubin and biliverdin,^14^ 2-(1′H-indole-3′-carbonyl)-thiazole-4-carboxylic acid methyl ester (ITE),^15^ indoxyl-3-sulfate (I3S), indole-3-carbinol (I3C), gallic acid,^16^ prostaglandins and eicosanoids.^17^ Additional AHR agonists are produced by the metabolism of commensal microorganisms.^9,18,19^ Moreover, some medications are reported to activate AHR, including omeprazole,^20^ sulindac,^21^ laquinimod,^22^ tapinarof,^23^ and diclofenac.^24^ The ability of AHR to interact with multiple molecules and its broad expression enables it to modulate diverse physiological processes in response to environmental, microbial and metabolic cues. In this review, we discuss the role of AHR in the immune response, with a focus on the gut–brain axis.^25^

**Table 1:** AHR ligands

Compound	Activity	Source
1,4-dihydroxy-2-naphthoic acid (DHNA)	Agonist	Microbial metabolism
2-(19H-indole-39-carbonyl)-thiazole-4-carboxylic acid methyl ester (ITE)	Agonist	Tryptophan metabolism
2-(Indol-3-ylmethyl)-3,39-diindolylmethane (Ltr-1)	Agonist	Cruciferous vegetables
2,3,7,8-Tetrachlorodibenzop-dioxin (TCDD)	Agonist	Anthropogenic
3,3-diindolylmethane (DIM)	Agonist	Cruciferous vegetables
3′4′-Dimethoxyflavone (DMF)	Partial agonist	Anthropogenic
3-Methylcholanthrene	Agonist	Anthropogenic
3-Methylindole (Skatole)	Partial agonist	Tryptophan metabolism
4-hydroxy-tamoxifen (4OHT)	Agonist	Anthropogenic
5-hydroxytryptophan (5HTP)	Agonist	Natural amino acid
6-Formylindolo [3,2-b] carbazole (FICZ)	Agonist	Tryptophan metabolism
6-Methyl-1,3,8-trichlorodibenzofuran (6-MCDF)	Partial agonist	Anthropogenic
Baicalin	Antagonist	*Scutellaria baicalensis* (plant)
Beta-naphthoflavone	Agonist	Anthropogenic
Bilirubin	Agonist	Heme metabolism
Biliverdin	Agonist	Heme metabolism
CH-22319	Antagonist	Anthropogenic
Cinnabarinic acid (CA)	Agonist	Tryptophan metabolism
Curcumin	Agonist	Natural pigment
Diclofenac	Agonist	Anthropogenic
Diosmin	Agonist	Citrus fruit peel
Gallic acid	Agonist	Diferent plants
GNF351	Antagonist	Anthropogenic
Hydroxyeicosatrienoic acid ([12(R)-HETE])	Agonist	Arachdonic acid metabolism
Indigo	Agonist	*Indigofera* spp (plant)
Indirubin	Agonist	*Indigofera* spp (plant)
Indole	Agonist	Diverse natural origen
Indole-3-acetic acid (IAA)	Agonist	Plant hormone (Indole derivative)
Indole-3-acetonitrile (I3ACN)	Agonist	Plant hormone (indole derivative)
Indole-3-aldehyde (IAId)	Agonist	Tryptophan metabolism by bacterias
Indole-3-carbinol (I3C)	Agonist	Cruciferous vegetables
Indolo [3,2-b]carbazole (ICZ)	Agonist	Indole-3-carbinol
Indoxyl-3-sulfate (I3S)	Agonist	Tryptophan metabolism
Kynurenic acid (KA)	Agonist	Tryptophan metabolism
l-Kynurenine (Kyn)	Agonist	Tryptophan metabolism
Laquinimod	Agonist	Anthropogenic
Lipoxin A4	Agonist	Arachidonic acid metabolism
Malassezin	Agonist	*Malassezia furfur* (fungi)
3′-methoxy-4′- nitroflavone (MNF)	Antagonist	Shynthetic falvone derivative
Norisoboldine	Agonist	*Lindera aggregata* (plant)
Omeprazole	Agonist	Anthropogenic
Prostaglandin	Agonist	Arachidonic acid metabolism
Quercetin	Partial agonist	Fruits and vegetables
StemRegenin 1 (SR1)	Antagonist	Purine derivative
Sulindac	Agonist	Anthropogenic
Resveratrol	Partial agonist	Fruits and vegetables
Tapinarof	Agonist	Bacterial metabolism
Tryptamine	Agonist	Tryptophan metabolism
Trypthantrin	Agonist	Tryptophan metabolism
VAF347	Agonist	Anthropogenic
Xanthurenic acid	Agonist	Tryptophan metabolism

## The AHR

**Fig. 1:**
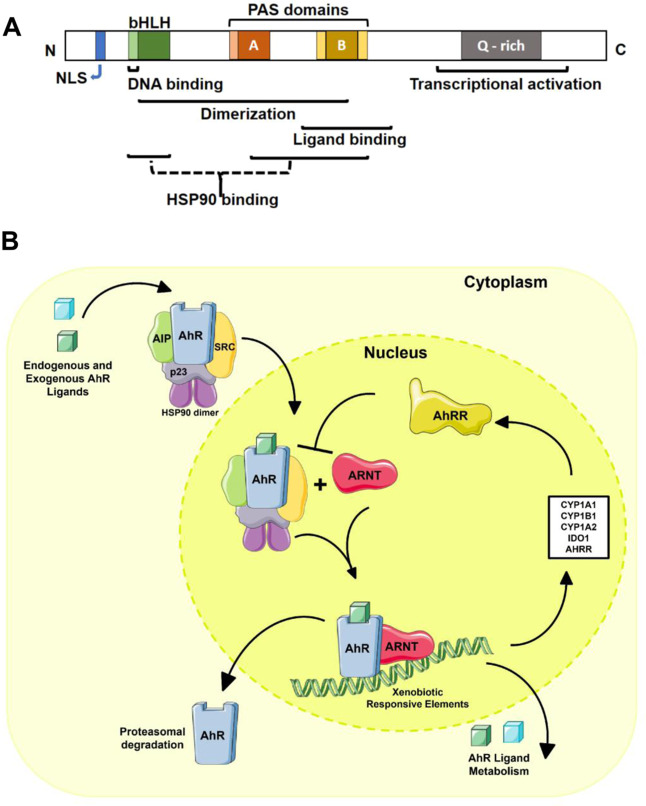
AHR and its signaling pathway. **A** Schematic representation of AHR protein domains. **B** The AHR signaling pathway. The inactive form of AHR is localized in the cytosol in a complex composed of HSP90, AIP, p23, and c-SRC. AHR agonists induce conformational changes in AHR that result in its translocation to the nucleus. In the nucleus, AHR interacts with ARNT, and the heterodimer is responsible for the transcription of XRE-containing genes. Notes: (AHR) aryl hydrocarbon receptor, (N) N terminal motif, (C) C terminal motif, (NLS) nuclear localization signal, (bHLH) basic-helix loop helix, (PAS) Per-Arnt-Sim, (Q-rich) glutamine rich, (HSP90) heat shock protein 90, (AIP) AHR-interacting protein, (XRE) xenobiotic responsive elements, (AHRR) AHR repressor, (CYP) cytochrome P450, (IDO) indoleamine 2,3-dioxygenase

In mice and humans, AHR is an 848-amino acid-long protein. It is encoded by a gene located on chromosomes 7 and 12 in humans^26^ and mice,^27^ respectively. The *Ahr* promoter harbors several transcription initiation sites rich in GC-rich regions but without a TATA or a CCAAT box.^28^ These GC-rich regions contain binding sites for ubiquitously expressed zinc-finger transcription factors, including Sp1 and Sp3, which seem to be required for basal *AHR* expression.^29^ AHR is a member of the basic-helix/loop/helix *per-Arnt-sim* (bHLH/PAS) family of transcription factors. The bHLH domain of AHR is responsible for DNA binding and dimerization, stabilizing protein–protein interactions. The PAS domain contains two subdomains: PAS-A, which is essential for dimerization with other proteins, and PAS-B, which harbors ligand- and heat shock protein (HSP) 90-binding motifs (Fig. 1A). The AHR transcriptional activation domain is located in the N terminal region and encompasses a region rich in glutamine (Q-rich region) that also harbors a nuclear translocation signal^30,31^ (Fig. 1A).

Inactive AHR is located in the cytoplasm complexed with several chaperones that stabilize it. The cytoplasmic AHR complex contains the following: (1) an HSP90 dimer that maintains AHR in a conformation that maximizes its affinity for ligands;^32^ (2) p23 as a cochaperone; (3) AHR-interacting protein (AIP, also known as Ara9 or XAP2), which stabilizes AHR in the cytoplasm, preventing its ubiquitination and degradation;^33^ and (4) the c-SRC protein kinase.^34^ AHR *genomic signaling* is triggered by ligand binding, which induces a conformational change in AHR, releasing AIP^35^ and exposing the nuclear localization signal^36^ and a protein kinase C target site that when phosphorylated promotes AHR nuclear translocation^37^ (Fig. 1B). HSP90 is reported to translocate to the nucleus together with AHR,^38^ but knowledge of the cofactors that translocate to the nucleus together with AHR and their function remains limited.

In the nucleus, AHR exchanges its chaperones with the AHR nuclear translocator (ARNT, also known as HIF-1β)^39^ to interact with DNA sequences known as xenobiotic responsive elements (XRE, also known as DRE) in the regulatory regions of target genes (e.g., *CYP1A1*, *CYP1A2*, *CYP1B1*, and *AHRR*). AHR-targeted components of cytochrome P450 (CYP) affected by AHR catalyze the degradation of AHR ligands^40^ and hence participate in a negative feedback loop that limits AHR activation. AHR also induces expression of the AHR repressor (AHRR), which limits AHR activation.^41^

In addition to its direct effects mediated via XREs, AHR controls transcriptional responses through interaction with other transcription factors and coactivators, including nuclear factor-*kappa*B (NF-κB), estrogen receptor (ER), retinoic acid receptor, and members of the signal transducers and activators of transcription family.^42^ For instance, NF-kB/RelA-dependent AHR expression following LPS stimulation in dendritic cells (DCs) has been described.^43^ AHR has also been shown to interact with c-Maf to control the expression of IL-10 and IL-21.^44–47^

AHR has been shown to modulate the epigenetic status of the cell via the control of noncoding RNAs,^48^ microRNAs,^49^ and histone acetylation/methylation mechanisms that regulate chromatin conformation and accessibility.^50^

AHR signaling also involves *nongenomic pathways*. For example, once released from its complex with AHR, c-SRC can phosphorylate enzymes involved in the arachidonic acid and leukotriene signaling pathways.^51^ These nongenomic mechanisms are important for the induction of endotoxin tolerance in DCs via c-SRC-driven phosphorylation and stabilization of indoleamine 2,3-dioxygenase 1 (IDO1).^52^ Finally, AHR has been reported to act as an E3 ubiquitin protein ligase, inducing proteasomal degradation of protein targets such as p53, FOS, hypoxia-inducible factor (HIF)-1α, MYC, and ER.^53–55^ Altogether, these data demonstrate that almost half a century after the cloning of AHR, the mechanisms mediating the control of cellular responses by AHR still need to be fully elucidated.

## AHR in the control of the immune response

The purification of AHR and the generation of knockout mice (AHR KO) led to the identification of multiple physiologic roles for AHR.^56,57^ One of those roles is the regulation of the immune response.

### Modulation of inflammation by AHR expressed in mucosal tissues and skin

The intestinal epithelium interacts with a myriad of microbial metabolites, pollutants, and dietary molecules. AHR acts as a sensor for many of these environmental stimuli, mediating some of their effects on the immune response. AHR conditional knockout mice generated using an intestinal epithelial cell (IEC)-specific Cre recombinase (Vil1-Cre) show increased susceptibility to *Citrobacter rodentium* infection.^58^ In addition, these mice display defective differentiation of intestinal stem cells, resulting in the malignant transformation of IECs.^58^ Conversely, AHR activation by dietary ligands limits intestinal stem cell proliferation by regulating the E3 ubiquitin ligases Rnf43 and Znrf3, suppressing Wnt/*β*-Catenin signaling. Within the context of inflammation, IFN-γ induces the expression of IDO1, which produces the AHR agonist L-kynurenine, thereby triggering AHR-driven upregulation of IL-10 receptor 1 and consequently amplifying the anti-inflammatory effects of IL-10.^59^ These findings highlight the anti-inflammatory role of AHR in IECs and its contribution to the integrity of the intestinal barrier.

AHR also controls the expression of antimicrobial peptides that fight pathogens in the gut. For instance, regenerating islet-derived protein III (REGIII)β and REGIIIγ are upregulated following administration of the probiotic-derived AHR agonist 1,4-dihydroxy-2-naphthoic acid (DHNA), altering the microbiome and ameliorating dextran sodium sulfate-induced colitis in mice.^60^ In support of a role for AHR in therapeutic interventions already in use, the increase in Th22 cell differentiation and IL-22 production induced by TNF blockade was abrogated by AHR inhibition in Crohn’s disease (CD) patients treated with antitumor necrosis factor (TNF)-blocking antibodies. These data suggest a new role for AHR agonists as potential adjuvants for anti-TNF therapy in CD patients.^61^

Although these and additional studies^40,47,58,62–69^ support a protective role for AHR in intestinal inflammation, some studies have challenged this notion. In particular, it was recently reported that environmental oxazoles induce the production of AHR agonists by IDO1 expressed in IECs and other cell types,^70^ which surprisingly leads to increased intestinal inflammation via the suppression of IL-10 secretion, modulation of CD1d-dependent antigen presentation and production of IFN*γ*/IL-13 by NKT cells.^70^ These provocative findings should be further investigated, particularly within the context of alternative interpretations, such as the expansion of AHR-driven nonpathogenic Th17 cells, which may acquire full pathogenic activity following exposure to additional factors in the inflamed gut, such as IL-23.^71^

Innate lymphoid cells (ILCs) are tissue-resident innate immune cells that participate in the response to infection and contribute to tissue homeostasis and chronic inflammation.^72,73^ ILCs are classified into five subsets: NK cells, lymphoid tissue inducer cells, group 1 ILCs (ILC1s), group 2 ILCs (ILC2s), and group 3 ILCs (ILC3s).^74^ Each of these subsets is controlled by different transcription factors,^75^ and AHR controls IL-22 expression in ILC3s.^76^ Indeed, AHR-deficient mice exhibit expansion of segmented filamentous bacteria in the small intestine due to reduced IL-22 production by ILC3s, which in turn promotes Th17 cell expansion in the gut and the development of spontaneous colitis.^72,73^ Of note, polymorphisms in caspase recruitment domain family member 9 (CARD9) have been associated with intestinal inflammation.^77^ Interestingly, CARD9 risk alleles associated with inflammatory bowel disease promote a decrease in the abundance of intestinal commensals that produce AHR agonists, leading to decreased AHR activation and intestinal inflammation.^77^ These findings highlight how AHR’s role as a sensor of commensal products contributes to mechanisms of intestinal pathogenesis.

DCs play central roles in the maintenance of tolerance and the generation of protective immune responses against pathogens in the gut as well as in other tissues.^78,79^ AHR is highly expressed in DCs,^80^ affecting their differentiation and function.^9,81^ AHR-driven cytokine, kynurenine,^82–84^ and retinoic acid^85^ production in DCs boosts the differentiation of regulatory T cells that suppress the development of experimental autoimmune encephalomyelitis (EAE), the model of multiple sclerosis (MS).^86^

Different subsets of DCs sense the lumen microenvironment, and following their migration to mesenteric lymph nodes (MLNs) via CCR7 signaling, they control peripheral differentiation regulatory cells and prime effector T cells.^87^ For example, AHR activation by the commensal metabolite indole-3-pyruvic acid reduces the ability of DCs in MLNs to promote the differentiation of IFN-γ-producing T cells, thus preventing chronic inflammation during colitis.^88^ AHR signaling in DCs is also reported to affect nonimmune cells in unexpected ways, as demonstrated by reports of increased numbers of small intestinal epithelial stem cells and atypical differentiation of epithelial precursors following AHR deletion from CD11c^+^ DCs.^89^

AHR signaling controls T-cell responses not only via the modulation of APC function but also through intrinsic effects in T cells. For example, AHR modulates the expansion and differentiation of Th17 cells,^90,91^ though AHR appears to be more relevant for the control of the transcriptional program of nonpathogenic Th17 cells.^71^ Indeed, AHR signaling promotes the conversion of Th17 cells to type 1 regulatory T cells (Tr1 cells).^92^ Moreover, AHR has been linked to the control of regulatory T cells through multiple mechanisms involving their differentiation and stability as well as effector mechanisms.^44,46,63,85,90,93^ Overall, the effects of AHR on T cells are likely to have consequences for inflammation in other tissues in addition to the gut.

Intraepithelial lymphocytes (IELs) constitute a population of T cells localized in the epithelial layer of mammalian mucosal linings such as the intestine. IELs are antigen-experienced T cells of both T-cell receptor γδ (TCRγδ)^+^ and TCRαβ^+^ lineages.^94^ AHR modulates IEL survival and response to nutritional and microbial stimuli.^95^ For example, administration of the AHR agonist FICZ ameliorates DDS-induced colitis by reducing the apoptotic rate of CD8αα^+^TCRαβ^+^ IELs, while decreasing and increasing their production of IFN-γ and IL-10, respectively.^96^ Furthermore, Colonna and collaborators established that CD8αα^+^TCRαβ^+^ IELs in the small intestine are supported by the activation of AHR signaling by tryptophan metabolites produced by *Lactobacillus reuteri*.^65^ Interestingly, Kadowaki et al. reported that microbial AHR agonists promote the differentiation of regulatory CD4^+^ IELs, which can migrate to the CNS and suppress inflammation through LAG3-dependent mechanisms.^97^ These important findings suggest that AHR-dependent immunoregulatory mechanisms operating in the gut can affect inflammatory processes in other tissues.

### Modulation of inflammation in the central nervous system (CNS)

The gut and brain axis is now recognized as a key factor in the pathology of multiple neurological disorders, including MS and its experimental model EAE.^25^ In EAE and MS, CD4^+^ effector T cells primed in the periphery migrate to the CNS, where they are reactivated by cDCs and other cells to cause myelin destruction.^98,99^ In addition, recruited T cells secrete cytokines that modulate the activity of CNS-resident immune cells, such as microglia and astrocytes.^100–102^

In pioneering studies, Wekerle and coworkers demonstrated that the commensal gut flora controls autoreactive T cells that migrate to the CNS and cause inflammation and tissue pathology.^103^ Follow-up studies defined alterations in the gut microbiota associated with MS^104^ and identified specific components of the microbiome involved in the regulation of effector and regulatory T cells.^105–107^ Similarly, it was recently reported that anti-inflammatory B cells controlled by the commensal flora migrate the gut to the CNS to limit tissue pathology in MS.^108^ Interestingly, AHR controls B-cell anti-inflammatory activities.^109,110^ Moreover, AHR controls the differentiation and stability of intestinal Tregs,^47,85^ and oral administration of the AHR agonist ITE increases the myelin-reactive Treg/Teff ratio and suppresses EAE.^85^ These findings suggest that AHR signaling contributes to the anti-inflammatory effects of the commensal flora not only in the gut but also in other tissues, such as the CNS.

Astrocytes are the most abundant glial cells in the CNS and have essential roles associated with the support of neurons and synapses, the control of neurotransmitters and the regulation of blood–brain barrier development and function.^111–118^ Astrocytes also play important roles in CNS inflammation and neurodegeneration via their own neurotoxic activities as well as the recruitment and activation of other cells involved in CNS pathogenesis.^19,100,119–126^ Transcriptional analyses of astrocytes revealed AHR upregulation in the context of EAE and MS.^119,121^ Indeed, specific inactivation of AHR in astrocytes via conditional knockout mice and cell-specific shRNA knockdown identified AHR as a negative regulator of the NF-κB transcriptional responses that promote microglial activation, neurotoxic peripheral monocyte recruitment to the CNS, and astrocyte-intrinsic neurotoxic activities.^19^ Moreover, in-depth molecular studies have established that AHR inhibits NF-κB activation in astrocytes through a SOCS2-dependent mechanism^19^ that also operates in DCs.^127^ Interestingly, microbiota perturbation studies showed that metabolites produced from the degradation of tryptophan by the intestinal commensal flora reach the CNS and activate AHR in astrocytes to limit CNS inflammation,^19^ describing for the first time a mechanism mediating the control of astrocytes by the gut flora.

Microglia are CNS-resident macrophages with multiple functions in health and disease,^128^ playing important roles in the control of astrocyte responses.^129^ Interestingly, microglia express AHR,^130,131^ and conditional knockout mice revealed that AHR limits NF-κB activation in microglia.^19^ In addition, AHR controls microglial production of TGF-α and VEGF-B: AHR transactivates the *Tgfa* promoter, interfering with NF-κB-driven VEGF-B expression.^19^ Microglial TGF-α and VEGF-B suppress and induce astrocyte responses, respectively, that promote CNS pathogenesis.^19^ In fact, deletion of microglial AHR worsens EAE, increasing demyelination and monocyte recruitment to the CNS.^19^ As microbial agonists can also activate microglial AHR, these findings provide a molecular mechanism by which the gut microbiome modulates microglial and astrocyte responses as well as interactions between these CNS-resident cells.

AHR is expressed in CNS endothelial cells,^132^ neurons,^133^ and oligodendrocytes.^134^ Endothelial cell AHR is suggested to contribute to detoxification processes^132,135,136^ and studies in fish suggest that AHR hyperactivation in endothelial cells trigger apoptosis and vascular defects, resulting in hemorrhage, edema, and embryonic mortality.^132^ Metabolites of the pesticide DDT induce AHR-dependent neurotoxicity.^137^ Finally, AHR has been proposed to participate in oligodendrocyte differentiation.^138^ These findings indicate that AHR participates in the regulation of endothelial cells, neurons and oligodendrocytes in health and disease, though further studies are needed to identify the specific mechanisms involved.

As mentioned above, AHR signaling can modulate peripheral T-cell differentiation.^9,81^ Moreover, peripheral T cells recruited to the CNS control astrocyte^100,101,120^ and microglial^102^ responses. Hence, these findings suggest that AHR signaling participates in the gut–brain axis through multiple mechanisms ranging from activation of AHR in CNS-resident cells by microbial metabolites to AHR-mediated peripheral modulation of immune cells that migrate to the CNS.

## Role of AHR in infections targeting the CNS

**Fig. 2:**
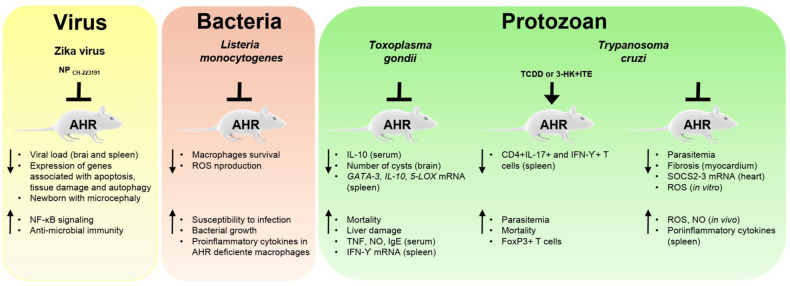
Role of AHR in infections targeting the CNS. AHR can affect the outcome of infectious diseases that target the CNS. Notes: (NP) nanoparticles, (ROS) reactive oxygen species, (NO) nitric oxide

The microbiota establishes multiple types of relationships with the host, ranging from mutualism to parasitism: in the former, the interaction is beneficial for both organisms; in the latter, this interaction is only beneficial for the parasite and harmful for the host.^139^ We discussed AHR-mediated microbiota–host relationships beneficial for the host above; below, we describe the role of AHR in relationships detrimental to the host (Fig. 2).

*Lysteria monocitogenesis* targets the gastrointestinal tract and can also cause meningitis. In a murine model of listeriosis, AHR-deficient mice displayed higher mortality than their WT counterparts, concomitant with higher levels of pro-inflammatory cytokines, decreased ROS production and macrophage survival.^140^ Zika virus (ZIKV) infection has been associated with severe outcomes, including fetal brain abnormalities^141^ and Guillain–Barré syndrome.^142^ Similarly, it was recently reported that ZIKV infection triggers the production of AHR agonists by the host.^126^ AHR activation interferes with IFN-I-dependent mechanisms of anti-ZIKV immunity,^126^ in agreement with previous reports.^143^ AHR also interferes with mechanisms of intrinsic immunity mediated by the protein PML. Most importantly, AHR inhibition with clinical antagonists suppresses ZIKV replication in vitro and in vivo and ameliorates CNS abnormalities associated with ZIKV.^126^ Similar mechanisms appear to operate within the context of infection by dengue virus.^126^

*Trypanosoma cruzi* is the etiological agent of Chagas’s disease, a chronic illness endemic to Central and South America with long-term consequences for the heart, esophagus, colon and nervous system.^144^ In the experimental model of Chagas disease, AHR activation expands the Treg compartment, increasing parasite replication.^145^ In agreement with these findings, AHR-deficient mice show reduced *T. cruzi* parasitemia and a heightened immune response characterized by the production of proinflammatory cytokines, increased NO in serum, and downregulation of *SOCS2*.^145,146^ Conversely, within the context of infection by *Toxoplasma gondii*, AHR deficiency results in higher mortality as a result of increased pro-inflammatory responses and decreased IL-10 production.^147^ Taken together, these findings highlight the complex roles played by AHR in infection: AHR can limit immunopathology but can also be exploited by pathogens to evade the immune response.

## AHR and CNS tumors

**Fig. 3:**
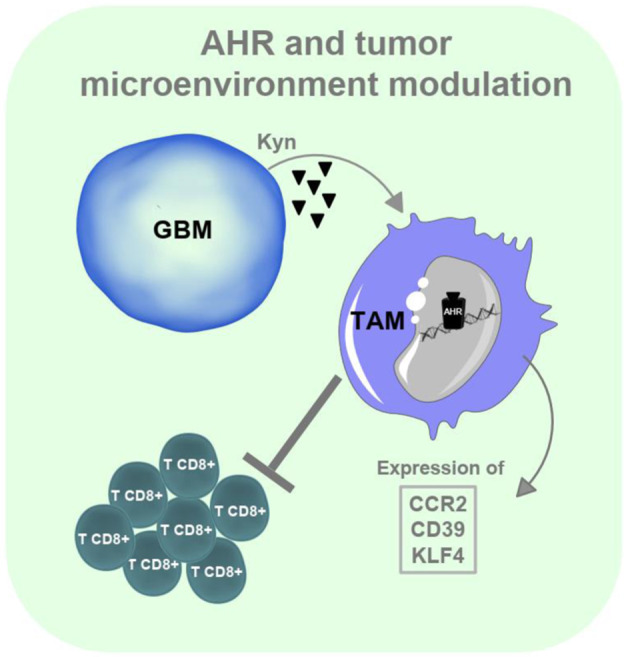
Role of AHR in glioblastoma. Kynurenine in the tumor microenvironment activates AHR in TAMs, promoting expression of CCR2, CD39 and KLF4. CCR2 contributes to the recruitment of TAMs to the tumor microenvironment, CD39 promotes CD8^+^ T-cell dysfunction, and KLF4 together with SOCS2 influences TAM polarization. Notes: (Kyn) kynurenine, (TAM) tumor-associated macrophages

Based on its multiple physiological roles, it is not surprising that AHR contributes to tumor pathogenesis. Glioblastoma is the most common primary malignant brain tumor in adults^148^ and one of the most aggressive cancers, with a median survival of 15–18 months despite standard of care therapy.^148,149^ Opitz et al. reported that tryptophan 2,3-dioxygenase in glioblastoma leads to the production of kynurenine, which acts in an autocrine manner to enhance tumor invasiveness and replication.^150,151^ In addition, Gramarzki et al. reported that AHR in glioma cells drives expression of TGF-β, suggesting that AHR signaling promotes an immune suppressive microenvironment in glioma.^152^ Indeed, AHR expression has been detected in tumor-associated macrophages (TAMs), which constitute more than 30% of infiltrating cells in glioblastoma. Takenaka et al. recently showed that AHR activation induces an anti-inflammatory phenotype in glioblastoma TAMs.^153^ Moreover, AHR drives expression of CD39 in TAMs, which promotes CD8^+^ cell dysfunction^153^ (Fig. 3). These findings suggest a role for AHR in tumor immunoevasion and highlight the intrinsic tumor cell functions, emphasizing its potential as a therapeutic target.^151,154,155^

## AHR as a target for therapeutic immunomodulation

**Fig. 4:**
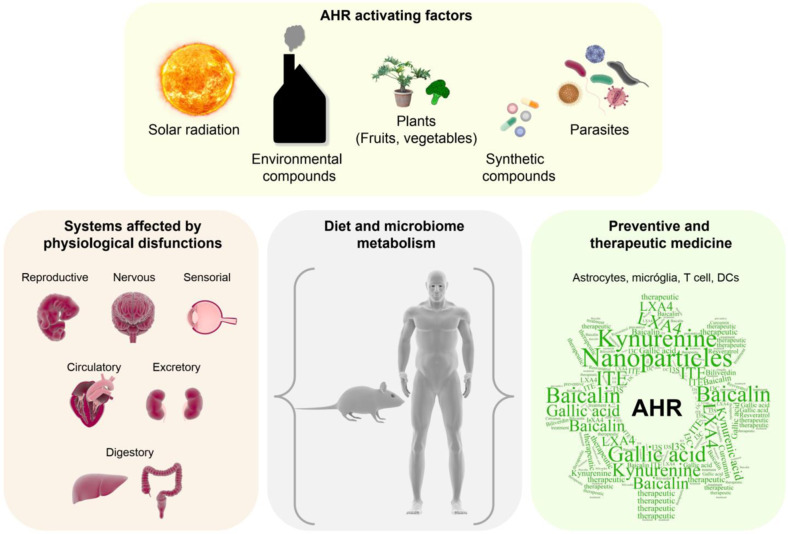
AHR sensor and immunomodulatory roles. AHR senses diverse environmental cues provided by the diet, microbiome, and anthropogenic compounds. AHR signaling participates in physiological and pathological processes, making it a potential target for therapeutic intervention

As briefly discussed in this manuscript, AHR signaling has multiple effects on the immune response. AHR constitutes a potential target for therapeutic intervention based on the ability of small molecules to control its activity (Fig. 4).

With regard to autoimmune diseases, laquinimod^22^ and tapinarof^23,156,157^ have been developed as AHR-targeting drugs for the treatment of MS, psoriasis and atopic dermatitis. Furthermore, codelivery of tolerogenic AHR agonists and antigens to DCs with nanoparticles provides an attractive approach. This nanoparticle-based approach is based on the induction of a tolerogenic phenotype in DCs, which are concomitantly loaded with disease-relevant antigens,^158^ thereby boosting antigen-specific tolerance with minimal effects on nonrelated immune responses. This approach leads to expansion of Tregs (both FoxP3 + Tregs and Tr1 cells) that suppress inflammation in EAE.^159^ Similar observations have been made in other autoimmune diseases, such as type 1 diabetes.^127^ Within the context of infection or tumors, AHR inhibitors may offer a novel pathway to limit immune evasion,^151^ with the caveat that AHR may also play a role in limiting immunopathology. Nonetheless, in considering the therapeutic targeting of AHR, it should be kept in mind that AHR participates in multiple physiological processes in addition to immune regulation. Moreover, AHR signaling is regulated by microbial metabolites, with important effects on the immune response. Thus, therapeutic targeting of AHR should consider not only its effects on the immune response but also its important roles in the host–microbiome relationship and the multiple effects of the microbiome in autoimmunity, cancer, and infections.

## Concluding remarks

Five decades after its identification, AHR has emerged as an important immune regulator. It is therefore important to characterize the physiological AHR agonists involved in immune regulation, as they may provide lead molecules for the development of novel immunomodulators. In addition, they may contribute to the identification of ligand-specific downstream effects of AHR signaling of therapeutic interest. Within this context, there remains an important need to characterize the cell-specific effects of AHR signaling and the mechanisms involved.

Finally, the participation of AHR in the gut–brain axis prompts new research questions, as follows: (1) Which microbial AHR agonists reach the CNS? (2) Which components of the commensal flora produce AHR ligands, and how are they regulated in health and disease? (3) Which peripheral cells are educated by the commensal flora in the periphery to then migrate to the CNS and control the activity of resident cells? These questions will guide future research efforts and reveal novel opportunities for AHR-targeted therapeutics.
